# Performance of a Vision-Language Model in Detecting Common Dental Conditions on Panoramic Radiographs Using Different Tooth Numbering Systems

**DOI:** 10.3390/diagnostics15182315

**Published:** 2025-09-12

**Authors:** Zekai Liu, Qi Yong H. Ai, Andy Wai Kan Yeung, Ray Tanaka, Andrew Nalley, Kuo Feng Hung

**Affiliations:** 1 Oral and Maxillofacial Radiology, Applied Oral Sciences and Community Dental Care, Faculty of Dentistry, The University of Hong Kong, Hong Kong SAR 999077, China; u3631841@connect.hku.hk (Z.L.); ndyeung@hku.hk (A.W.K.Y.); rayt3@hku.hk (R.T.); 2 Department of Diagnostic Radiology, Li Ka Shing Faculty of Medicine, The University of Hong Kong, Hong Kong SAR 999077, China; hemisai@hku.hk

**Keywords:** artificial intelligence, vision-language models, large language models, panoramic radiographs, diagnostic accuracy, dentistry

## Abstract

**Objectives**: The aim of this study was to evaluate the performance of GPT-4o in identifying nine common dental conditions on panoramic radiographs, both overall and at specific tooth sites, and to assess whether the use of different tooth numbering systems (FDI and Universal) in prompts would affect its diagnostic accuracy. **Methods**: Fifty panoramic radiographs exhibiting various common dental conditions including missing teeth, impacted teeth, caries, endodontically treated teeth, teeth with restorations, periapical lesions, periodontal bone loss, tooth fractures, cracks, retained roots, dental implants, osteolytic lesions, and osteosclerosis were included. Each image was evaluated twice by GPT-4o in May 2025, using structured prompts based on either the FDI or Universal tooth numbering system, to identify the presence of these conditions at specific tooth sites or regions. GPT-4o responses were compared to a consensus reference standard established by an oral-maxillofacial radiology team. GPT-4o’s performance was evaluated using balanced accuracy, sensitivity, specificity, and F1 score both at the patient and tooth levels. **Results**: A total of 100 GPT-4o responses were generated. At the patient level, balanced accuracy ranged from 46.25% to 98.83% (FDI) and 49.75% to 92.86% (Universal), with the highest accuracies for dental implants (92.86–98.83%). F1-scores and sensitivities were highest for implants, missing, and impacted teeth, but zero for caries, periapical lesions, and fractures. Specificity was generally high across conditions. Notable discrepancies were observed between patient- and tooth-level performance, especially for implants and restorations. GPT-4o’s performance was similar between using the two numbering systems. **Conclusions**: GPT-4o demonstrated superior performance in detecting dental implants and treated or restored teeth but inferior performance for caries, periapical lesions, and fractures. Diagnostic accuracy was higher at the patient level than at the tooth level, with similar performances for both numbering systems. Future studies with larger, more diverse datasets and multiple models are needed.

## 1. Introduction

With the rapid growth of technology, artificial intelligence (AI) models have become more involved in various aspects of daily life, including a wide range of medical and dental applications [1,2]. In dentistry, AI models has been proposed for tasks such as disease detection, segmentation, and prediction [3,4,5]. The advancement of AI research and its applications in these fields has been driven in large part by the development and availability of large, high-quality datasets [6,7,8,9,10,11,12,13,14]. Among recent AI innovations, large language models (LLMs) have emerged as powerful tools capable of understanding, generating, and using human language [15]. They learn to predict the next word in a sequence, allowing them to perform various language-related tasks like answering questions, summarizing text, translating languages, and even generating creative content [16]. Previous studies have introduced a cloud-based intelligent system built on LLMs to help patients with medical care and registration [17]. In addition, some studies have evaluated the performance of LLMs in completing licensing exams or generating radiology reports [18,19]. Several commercially available AI chatbots have shown the potential to perform at levels similar to clinicians, and they are providing valuable information for researchers [8,20].

Chat Generative Pre-trained Transformer (ChatGPT) has become one of the most popularly used LLMs since its release at the end of 2022 [21]. The early GPT versions could only process and interpret information through text and could not directly read images. Starting from GPT-4V, users can upload image files directly to the chat and have them interpreted instantly. Researchers have already evaluated GPT-4V’s performance in many dental fields, such as completing the national dental licensing exam and detecting supernumerary teeth [22,23]. With the release of GPT-4V, it opens up more opportunities for dental AI applications. Officially released on 31 August 2024, GPT-4o has a faster response time, lower resource demand, and better performance in various medical fields compared to GPT-3.5 or, in some cases, even resident physicians [24].

Panoramic radiography has played an important role in dental diagnostics. It provides a comprehensive view of the oral cavity, including teeth and bone structures [25]. Panoramic imaging is not only easily accessible in a wide range of dental settings compared to other advanced radiographic examinations, but it also exposes patients to relatively low levels of ionizing radiation [26]. These advantages make panoramic radiography a highly versatile and widely used imaging method in dental diagnostics [27]. Interpreting panoramic radiographs greatly depends on the clinicians’ experience and can be relatively challenging. Recent systematic reviews have summarized the potential of AI and LLMs in dental and maxillofacial imaging [28,29,30,31,32,33,34]. Previous studies have reported promising results for LLMs in generating radiology reports, detecting supernumerary teeth, and answering radiology-related questions [19,23,35]. However, the performance of GPT-4o in identifying common dental conditions on panoramic radiographs both overall and at specific tooth sites has not yet been evaluated, and the impact of different tooth numbering systems in prompts on its performance remains unknown. Therefore, the objectives of this study were to evaluate the performance of GPT-4o in identifying nine common dental conditions on panoramic radiographs, both overall and at specific tooth sites, and to assess whether the use of different tooth numbering systems (FDI and Universal) in prompts would affect its diagnostic accuracy.

## 2. Materials and Methods

### 2.1. Study Design

This observational study was designed to evaluate the performance of AI chatbots in detecting common dental conditions on panoramic radiographs. The study was conducted in full accordance with the Declaration of Helsinki 2013 (www.wma.net). The study protocol was submitted to and approved by the local institutional review board of the University of Hong Kong/Hospital Authority Hong Kong West Cluster (approval number UW 25-270).

### 2.2. Study Population

The primary objective of this pilot study was to evaluate the performance of GPT-4o in identifying various conditions, including caries, periapical lesions, periodontal bone loss, impacted third molars, restorations, implants, radiopaque, mixed-density, or radiolucent pathological jaw lesions, on panoramic radiographs. To ensure the inclusion of only images presenting the investigated conditions, this pilot study retrospectively retrieved 320 panoramic radiographs from patients who had undergone CBCT examinations between January 2020 and December 2022 in our Diagnostic Imaging Clinic, for dentoalveolar, endodontic, periodontal, dental implant evaluations with clinical records indicating the presence of the investigated conditions. Subsequently, two examiners, a Board-Certified Radiologist (AN) and a Professor in the Oral Radiology Department (KFH), reviewed the 320 panoramic images for inclusion. Panoramic radiographs were excluded if any of the following exclusion criteria were met: (1) patients < 18 years of age; (2) images not presenting at least one of the investigated conditions; (3) images where the presence of the investigated conditions was doubtful and consensus could not be reached between the examiners; or (4) images with severe positioning errors, ghost images, or motion artifacts. Afterwards, a total of 50 panoramic radiographs were included in this pilot study. The number of cases for each condition is provided in Table 1. All panoramic radiographs were acquired using the Veraview X800 (J. Morita, Kyoto, Japan) at the Diagnostic Imaging Clinic at Prince Philip Dental Hospital, Faculty of Dentistry, the University of Hong Kong.

### 2.3. Prompt Development and Response Generation

This study was conducted using GPT-4o accessed via the University of Hong Kong. Structured prompts were developed to evaluate GPT-4o’s capability in detecting common dental conditions on panoramic radiographs and to assess whether incorporating different tooth numbering systems (FDI or Universal) into the prompts would impact its performance. For each image, two prompts were used with the only difference being the tooth numbering system referenced. Each image was uploaded as a new file, and a new chat topic was initiated for every case to prevent data interruption and potential context contamination. If a response did not specify the exact tooth number, a follow-up question was issued requesting this information. All responses were generated in May 2025. The prompts provided clear instructions to focus solely on visible patterns and to avoid making clinical diagnoses. The prompts were as follows:

*FDI numbering system prompt:*
“This is an anonymized dental panoramic radiograph from a research study. You are assisting in identifying observable dental anomalies as listed below, and specify the FDI tooth number of identified items. Focus only on visual patterns, not diagnoses.
Developmental anomalies:

- Hypodontia (missing teeth) or anodontia.

Dental anomalies:

- Caries (radiolucent lesions in enamel/dentin).
- Periapical lesions (e.g., radiolucency at root apex indicating infection or granuloma).
- Tooth fractures, cracks, or retained roots.
- Impacted teeth (e.g., third molars, canines).

Bony anomalies:

- Periodontal bone loss (horizontal/vertical reduction in alveolar bone height).
- Osteolytic lesions (e.g., cysts, tumors like ameloblastoma or odontogenic keratocyst).
- Osteosclerosis (abnormal bone density, e.g., condensing osteitis).

Iatrogenic/treatment-related findings:

- Endodontically treated teeth or teeth with restorations (Fillings, Crowns or bridges).
- Dental implants (screw-shaped radiopaque structures).”

*Universal numbering system prompt:*
The second prompt was identical in structure and content, except that it instructed ChatGPT to specify the Universal tooth number for identified findings.

### 2.4. Data Collection

After collecting all the responses, the data were stored in an Excel spreadsheet (Microsoft, Redmond, DC, USA). In the Excel spreadsheet, all the investigated conditions were listed for each tooth. When GPT-4o indicated that there were conditions and specified the tooth number, a “positive” outcome was recorded for that specific tooth sites. Otherwise, any conditions that were not mentioned in the GPT-4o’s response were recorded as “negative”. Both the response using the FDI and the Universal numbering system were recorded in separate files. A postgraduate dental student in dento-maxillofacial radiology (ZL) independently generated all responses from GPT-4o using the prompts and recorded GPT-4o’s findings. The reference standard for the presence or absence of each dental condition was established by consensus among a postgraduate student, an oral-maxillofacial radiologist, and a professor in the Oral Radiology department. True positives, true negatives, false positives, and false negatives were then determined by comparing GPT’s outputs to this reference standard.

Panoramic images have reported accuracy levels of 70–85% for diagnosing periodontal bone loss [36,37]. In order to evaluate GPT-4o′s ability to correctly identify moderate to severe bone loss at the tooth-specific level, a case was considered positive for “periodontal bone loss” only if the alveolar bone level was located apical to the midpoint of the root.

For the “osteolytic lesions” and “osteosclerosis” conditions, their locations were assessed in six regions, including the anterior maxilla, anterior mandible, left posterior maxilla, right posterior maxilla, left posterior mandible, and right posterior mandible, rather than specific tooth sites.

### 2.5. Statistical Analysis

All collected data were initially analysed descriptively to summarize the distribution of positive and negative cases in each dental condition category. To provide a more reliable assessment of GPT-4o’s performance, the diagnostic performance of GPT-4o was evaluated for each condition and numbering system (FDI and Universal) using sensitivity, specificity, balanced accuracy (average of sensitivity and specificity), and F1-score (harmonic mean of precision and recall). These evaluation metrics were determined by comparing GPT-4o’s predictions to the reference standard using counts of true positives (TP), true negatives (TN), false positives (FP), and false negatives (FN) for each condition. For tooth-level analysis, each tooth or region was treated as an independent unit. For patient-level analysis, a patient was considered positive for a condition if GPT-4o correctly identified the condition on any tooth. Otherwise, the patient was considered negative. All statistical analyses were performed using SPSS software (Version 29.0; IBM Corp., Armonk, NY, USA).

## 3. Results

A total of 100 responses were recorded for fifty images. Table 1 shows the count of positive and negative cases for each dental condition at both the patient and tooth levels as identified by the reference standard and by GPT-4o using either the FDI or universal numbering system.

Table 2 demonstrates the diagnostic performance of GPT-4o, including balanced accuracy, sensitivity, specificity, and F1-score for each condition at both the patient and tooth levels.

At the patient level, balanced accuracy ranged from 46.25% to 98.83% with the FDI system and from 49.75% to 92.86% with the universal system. Tooth-level balanced accuracy was generally lower but more consistent, ranging from 49.66% to 65.04% (FDI) and 49.75% to 77.89% (universal). The highest patient-level balanced accuracies were observed for Dental Implants (98.83% FDI, 92.86% universal) and Endodontically Treated Teeth or Teeth with Restorations (80.92% FDI, 79.61% universal). At the tooth level, the highest balanced accuracies were found for Dental Implants (65.04% FDI, 77.89% universal), Impacted Teeth (61.35% FDI, 68.81% universal), and Missing Teeth (61.65% FDI, 63.96% universal).

F1-score showed considerable variability across conditions, with the highest scores at the patient level for Dental Implants (99.33% FDI, 92.31% universal), Endodontically Treated Teeth or Teeth with Restorations (89.19% FDI, 87.67% universal), and Missing Teeth (73.68% FDI, 77.92% universal). At the tooth level, F1-scores were generally lower, with the highest values for Missing Teeth (34.25% FDI, 38.69% universal), Impacted Teeth (49.52% FDI, 45.87% universal), and Dental Implants (28.57% FDI, 48% universal). Notably, several conditions exhibited an F1-score of 0 at both the patient and tooth levels, including Caries, Periapical Lesions, and Tooth Fractures/Cracks/Retained Roots.

Sensitivity at the patient level ranged from 0% to 100% (FDI) and 0% to 85.71% (universal), with the highest values for Dental Implants (100% FDI, 85.71% universal), Endodontically Treated Teeth or Teeth with Restorations (86.84% FDI, 84.21% universal), and Missing Teeth (80% FDI, 85.71% universal). At the tooth level, sensitivity was lower overall, ranging from 0% to 48.15% (FDI) and 0% to 46.30% (universal). The highest tooth-level sensitivities were for Impacted Teeth (48.15% FDI, 46.30% universal), Dental Implants (30.77% FDI, 46.15% universal), and Missing Teeth (28.72% FDI, 33.33% universal).

Specificity was generally high, particularly at the tooth level (mostly above 91%). At the patient level, the highest specificities were for Dental Implants (97.67% FDI, 100% universal), Osteolytic Lesions/Osteosclerosis (97.30% FDI/universal), and Caries (95.35% FDI, 97.67% universal). At the tooth level, Periapical Lesions (99.87% FDI, 100% universal), Caries (99.81% FDI, 99.87% universal), and Tooth Fractures, Cracks, or Retained Roots (99.56% FDI, 99.49% universal) had the highest specificities.

The largest discrepancies in balanced accuracy between patient and tooth levels were observed for Dental Implants (FDI: 33.8%, universal: 14.97%), Endodontically Treated Teeth or Teeth with Restorations (FDI: 20.7%, universal: 21.08%), and Missing Teeth (FDI: 15.0%, universal: 11.1%). In contrast, the smallest differences were found for Periodontal Bone Loss (FDI: 0.17%, universal: 0.59%), Impacted Teeth (FDI: 1.4%, universal: 3.75%), and Caries (FDI: 2.24%, universal: 1.10%).

**Figure 1:**
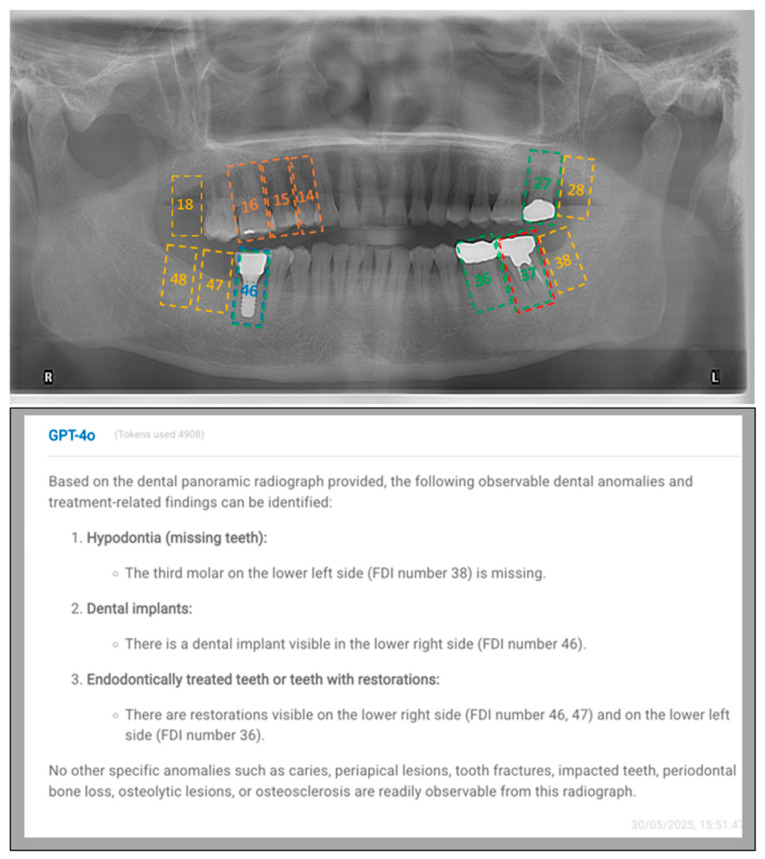
A representative case, displaying both the input image (**above**) and GPT-4o’s output (**below**). In this case, GPT-4o did not detect some dental conditions at the correct tooth sites. Teeth 18, 28, 38, 47, and 48 are missing (yellow doted rectangles) in this image, but only tooth 38 was identified as missing by GPT-4o. A dental implant is present at the site of tooth 46 (blue doted rectangle), which was accurately detected by GPT-4o. While GPT-4o correctly detected the presence of three teeth with restorations (green doted rectangles) in the posterior mandible, it misidentified their locations as 36, 46, and 47 instead of the correct sites, 36, 37, and 46. Neither the filling (orange doted rectangle) in teeth 14, 15, 16 nor the endodontic treatment (red doted rectangle) on tooth 37 was identified by GPT-4o.

**Figure 2:**
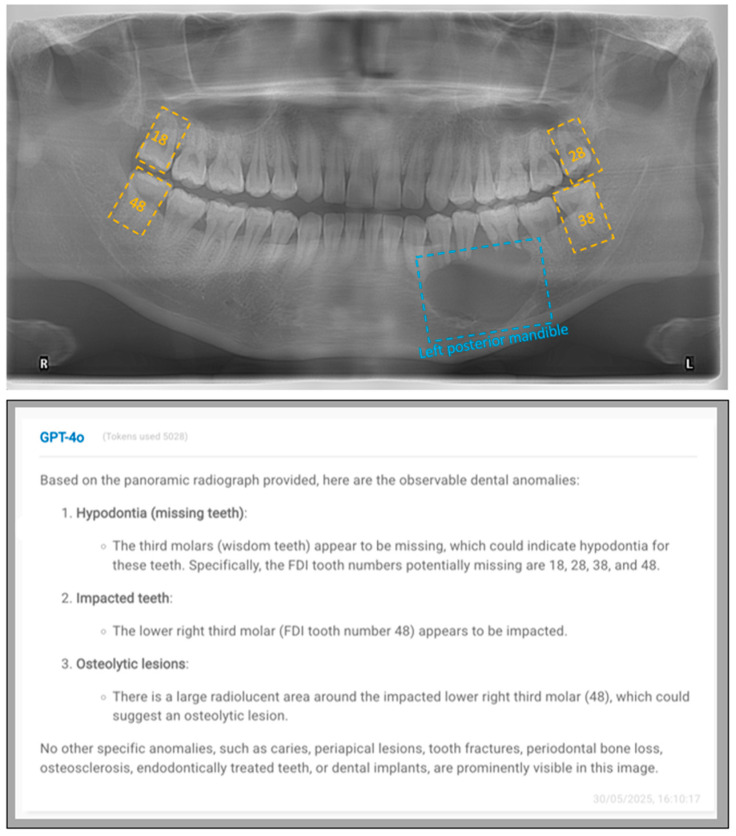
A representative case, displaying both the input image (**above**) and GPT-4o’s output (**below**). In this case, GPT-4o detected some dental conditions that are not present in the image, demonstrating hallucination. Although GPT-4o identified multiple missing teeth and a third molar (yellow doted rectangles), these findings are false positives, as all teeth are present in the image. GPT-4o correctly identified the presence of an osteolytic lesion in the mandible (blue doted rectangle), but mislocated it to the right posterior region instead of the left posterior region.

Figure 1 and Figure 2 exhibit representative cases to show both the input images and GPT-4o’s outputs.

## 4. Discussion

This study evaluated the performance of GPT-4o in identifying common dental conditions on panoramic radiographs, focusing on diagnostic accuracy both overall and at specific tooth sites. Additionally, it assessed whether using different tooth numbering systems (FDI and Universal) in prompts would affect GPT-4o’s diagnostic performance. The results showed that GPT-4o’s performance changed largely across different conditions and between patient and tooth levels. At the patient level, balanced accuracy ranged from 46.25% to 98.83% with the FDI system and from 49.75% to 92.86% with the *universal* system, while at the tooth level, balanced accuracy was generally lower but more consistent, ranging from 49.66% to 65.04% (FDI) and 49.75% to 77.89% (universal). Sensitivity demonstrated considerable variability, with higher values for certain conditions at the patient level, but generally lower and less variable results at the tooth level. In contrast, specificity was consistently high across most conditions, particularly at the tooth level, frequently exceeding 91%. F1-scores also reflected these trends, with higher values observed for some conditions at the patient level, but overall lower scores at the tooth level and several conditions exhibiting F1-scores of zero. These findings indicate that while GPT-4o achieved high specificity in excluding negative cases, its ability to consistently and accurately detect positive cases remains highly variable depending on the dental condition.

In this study, GPT-4o demonstrated the highest diagnostic performance for dental implants, endodontically treated teeth or teeth with restorations, and missing teeth, particularly at the patient level. Patient-level balanced accuracy was approximately 95% for dental implants and 80% for endodontically treated or restored teeth. GPT-4o successfully identified these conditions, which have radiopaque appearances and easily recognizable features on panoramic images, in most cases at the patient level. In contrast, GPT-4o’s performance was limited for caries, periapical lesions, and tooth fractures, cracks, or retained roots, with F1-scores and sensitivities of zero at both patient and tooth levels. These findings suggest that at the current stage GPT-4o is more capable of detecting radiopaque dental conditions than radiolucent ones, likely due to the distinct radiographic characteristics that make radiopaque entities more readily identifiable by AI models [38]. Previous studies have shown that AI models achieved high accuracy in detecting dental implants in panoramic radiographs [39,40]. On the contrary, more subtle radiolucent conditions (e.g., caries, periapical lesions, and tooth fractures) are relatively challenging to detect because of their less noticeable radiographic appearance and the limited detail provided by panoramic images. Zadrożny et al. found that while deep learning models obtained high sensitivity for dental fillings, endodontically treated teeth, residual roots, periodontal bone loss, missing teeth, and prosthetic restorations, these models showed low sensitivity for periapical lesions, caries, and over- and underfilled canals [41]. Similarly, Zhu et al. reported that the sensitivity and specificity of deep learning models for detecting caries in panoramic radiographs were not favourable. These findings suggest that AI-assisted diagnosis of subtle radiolucent lesions in panoramic images remains challenging [42].

In this study, large differences were found in GPT-4o’s performance between patient and tooth levels for certain conditions, particularly for dental implants, endodontically treated teeth or teeth with restorations, and missing teeth. For these conditions, the discrepancies in balanced accuracy between patient and tooth levels were considerable. The higher performance at the patient level indicates that GPT-4o could recognize the presence of these conditions in the image. However, it was often unable to accurately localize and assign the findings to the correct tooth sites, resulting in reduced tooth-level accuracy. This limitation in localization may be partially attributed to limitations of panoramic radiographs, such as overlapping anatomical structures, tooth crowding, and image distortion, which could affect GPT-4o’s ability in precise localization of specific teeth [43,44].

It was noted that the majority of the existing teeth misclassified as missing (i.e., false negative) were third molars. This finding suggests that GPT-4o had difficulty in accurately detecting third molars, which might be due to the high variability in their appearance and positioning on panoramic radiographs [45]. Third molars often present with inconsistent radiographic features owing to a wide range of anatomical variations and may be partially erupted, impacted, or superimposed by other anatomical structures, which makes them challenging for AI to identify [46]. Suárez et al. found that ChatGPT-4o frequently provided incomplete or fabricated information in complex third molar cases, particularly those involving overlapping anatomical structures or underdeveloped roots [47]. In this study, GPT-4o was observed to classify around fifteen third molars as both “missing teeth” and “impacted teeth”. This finding indicates that GPT-4o may consider third molars to be missing if they are impacted and not aligned at the same occlusal level as adjacent molars, which suggests a lack of clear understanding regarding the mutually exclusive nature of these conditions for the same tooth. Another study reported that GPT-4 performed poorly in detecting resorptive changes of impacted canines on panoramic radiographs, which suggests that accurately identifying changes in tooth structure changes remains challenging for commercially available LLMs [48].

While LLMs were expected to be able to detect large and well-defined radiopaque or radiolucent jaw lesions, in this study GPT-4o performed poorly in detecting the jaw lesions compared to its performance in identifying relatively less obvious conditions, such as periodontal bone loss. The sensitivity for osteolytic lesions or osteosclerosis was low at both the patient level (7.69%) and sextant level (3.33%) with F1-scores also near zero, which indicates that GPT-4o could not identify almost any true positive cases. This surprising outcome may be partially attributed to the prompt design without clear guidance for lesion detection. Notably, previous studies evaluating commercially available LLMs for radiographic image interpretation have rarely provided detailed descriptions of their prompt design, and those that did typically used a single prompting strategy rather than directly comparing multiple approaches. A recent study by Silva et al. evaluated a GPT model’s performance in describing radiolucent lesions in panoramic radiographs using a series of structured, stepwise prompts [49]. This approach first asked the model to provide a detailed radiographic description (covering internal structure, periphery, location, and effects on adjacent structures), then to offer impressions regarding the lesion’s nature, and finally to generate a list of likely differential diagnoses. Although this structured strategy gave clear direction for identifying both bony and dental anomalies, the reported accuracy in characterizing lesions remained limited, ranging from 25% to 67.85%, highlighting the ongoing challenges in applying LLMs for lesion detection and differential diagnosis in clinical practice. Based on previous studies, our study further assessed the potential of GPT in detecting common dental conditions using checklist-style prompts, which have not yet been investigated in previous studies. The prompting approach used in this study directed the model to search for specific anomalies, focusing mainly on visual patterns without requiring interpretative steps. This alternative approach may provide additional information regarding how prompt design may influence LLM performance in dental radiographic analysis.

Notably, for certain conditions such as caries and periapical lesions, GPT-4o obtained zero sensitivity but maintained high accuracy and specificity at both the patient and tooth levels. This phenomenon indicates that GPT-4o could not identify any true positive cases from the included images, resulting in a great amount of correctly classified negative cases. As the number of positive and negative cases for all conditions were relatively imbalanced at the tooth level, conventional evaluation metrics, such as accuracy and specificity, can be misleading, as high values may simply reflect the predominance of negative cases rather than true diagnostic capability. Instead of these metrics, sensitivity, balanced accuracy, and F1-score could provide a more reliable assessment of AI’s ability to correctly identify positive cases for analysis, with positive cases fewer than negative cases [50]. It is important to mention that GPT-4o was not specifically developed for medical or dental diagnostic purposes and should not be used for clinical decision-making, particularly in dental diagnostics. Our findings clearly demonstrate that GPT-4o could not detect subtle but clinically important radiolucent conditions at both the patient and tooth levels.

This study has several limitations. First, the sample size was relatively small, comprising only fifty images and nine common dental conditions, which may limit the generalizability of the findings. However, it is worth noting that the sample size in this study was greater than that of previous studies assessing the performance of ChatGPT in interpreting panoramic radiographs (which used between 28 and 30 images), and that each of the fifty images in this study was assessed twice using prompts with different tooth numbering systems, which was not evaluated in previous studies [47,49]. Moreover, this study did not evaluate other LLMs as GPT-4o was the only publicly accessible LLM with image reading capabilities (i.e., vision-language models) available during the data collection period. Future studies should include larger and more diverse datasets with a more balanced distribution of positive and negative cases at the tooth level. Additionally, the images selected in this study were deemed to have minimal imaging or patient positioning artifacts to reduce imaging quality, so that GPT-4o’s ability could be assessed from higher quality images. Furthermore, studies specifically designed to evaluate the performance of multiple LLMs across a wider range of dental conditions and image qualities, as well as to compare LLM performance with dental students and practitioners of different qualifications, will be needed to more comprehensively understand the potential and limitations of these models in dental diagnostics.

## 5. Conclusions

Based on the findings of this study, the following conclusions can be drawn:

- GPT-4o generally demonstrated low accuracy in identifying many of the investigated radiolucent conditions, with relatively higher accuracy only for easily recognizable radiopaque features such as dental implants, endodontically treated teeth, and teeth with restorations, indicating that it was unable to reliably detect subtle but clinically important radiolucent conditions at both the patient and tooth levels.
- GPT-4o achieved higher diagnostic performance at the patient level than at the tooth level for dental implants, endodontically treated teeth, and missing teeth, suggesting that while the model could detect the presence of these conditions in the image, it might not accurately localize them.
- GPT-4o demonstrated similar diagnostic performance when using either the FDI or Universal numbering systems in identifying most dental conditions.
- Future studies should use larger and more diverse datasets, evaluate the performance of multiple language models across a wider range of dental conditions and image qualities, and include comparisons with dental students and practitioners of varying qualifications to more comprehensively understand the potential and limitations of LLMs in dental diagnostics.

## Figures and Tables

**Table 1:** The count of positive and negative cases for each dental condition identified in the included images at both the patient and tooth levels as determined by the reference standard and by GPT-4o using either the FDI or universal numbering system.

Condition	Level	Positive Count (Reference Standard)	Negative Count (Reference Standard)	FDI Numbering System		Universal Numbering System	
				Positive Count (GPT-4o)	Negative Count (GPT-4o)	Positive Count (GPT-4o)	Negative Count (GPT-4o)
Missing teeth	Patient	35	15	41	9	42	8
	Tooth	195	1405	132	1468	141	1459
Impacted teeth	Patient	23	27	33	17	31	19
	Tooth	54	346	51	349	55	345
Caries	Patient	7	43	2	48	1	49
	Tooth	21	1579	3	1597	2	1598
Endodontically treated teeth or teeth with restorations (Fillings, Crowns or bridges)	Patient	38	12	36	14	35	15
	Tooth	202	1398	103	1497	110	1490
Periapical lesions	Patient	10	40	3	47	0	50
	Tooth	17	1583	2	1598	0	1600
Periodontal bone loss	Patient	23	27	8	42	5	45
	Tooth	305	1295	41	1559	28	1572
Tooth fractures, cracks, or retained roots	Patient	10	40	2	48	3	47
	Tooth	16	1584	7	1593	8	1592
Dental implants	Patient	7	43	8	42	6	44
	Tooth	13	1587	15	1585	12	1588
Osteolytic lesions or Osteosclerosis	Patient	13	37	2	48	2	48
	Region	14	295	2	307	2	309

**Table 2:** The diagnostic performance of GPT-4o for detecting each condition at both the patient and tooth levels using either the FDI or universal numbering system.

Condition	Level	F1-Score (%)		Balanced Accuracy (%)		Sensitivity (%)		Specificity (%)	
		FDI	Univ.	FDI	Univ.	FDI	Univ.	FDI	Univ.
Missing teeth	Patient	73.68	77.92	46.67	52.86	80	85.71	13.33	20
	Tooth	34.25	38.69	61.65	63.96	28.72	33.33	94.59	94.59
Impacted teeth	Patient	64.29	66.67	60	65.06	78.26	78.26	44.44	51.85
	Tooth	49.52	45.87	61.35	68.81	48.15	46.30	92.77	91.33
Caries	Patient	0	0	47.67	48.84	0	0	95.35	97.67
	Tooth	0	0	49.91	49.94	0	0	99.81	99.87
Endodontically treated teeth or teeth with restorations	Patient	89.19	87.67	80.92	79.61	86.84	84.21	75	75
	Tooth	32.13	28.21	60.2	58.53	24.26	21.78	96.14	95.28
Periapical lesions	Patient	0	0	46.25	50	0	0	92.5	100
	Tooth	0	0	49.94	50	0	0	99.87	100
Periodontal bone loss	Patient	25.81	21.43	51.29	52.82	17.39	13.04	85.19	92.59
	Tooth	8.67	9.76	51.46	52.23	4.92	5.32	97.99	99.15
Tooth fractures, cracks, or retained roots	Patient	0	15.38	47.5	52.5	0	10	95	95
	Tooth	0	0	49.77	49.75	0	0	99.56	99.49
Dental implants	Patient	99.33	92.31	98.83	92.86	100	85.71	97.67	100
	Tooth	28.57	48	65.04	77.89	30.77	46.15	99.31	99.62
Osteolytic lesions or Osteosclerosis	Patient	13.33	13.33	52.49	52.49	7.69	7.69	97.30	97.30
	Region	0	11.76	49.66	53.16	0	6.67	99.32	99.66

## Data Availability

Data will be provided by the corresponding author upon reasonable request.

## Abbreviations

The following abbreviations are used in this manuscript:

AI	Artificial intelligence
LLM	Large language model
ChatGPT	Chat Generative Pre-trained Transformer
